# Antibiotic stewardship: what for?

**DOI:** 10.3389/frabi.2025.1680329

**Published:** 2025-10-20

**Authors:** Carlos F. Amábile-Cuevas

**Affiliations:** Fundación Lusara, Mexico City, Mexico

**Keywords:** antibiotic stewardship, antibiotic resistance, antibiotic usage, socioeconomic factors, horizontal gene transfer

## Abstract

Antibiotic stewardship programs and controlled antibiotic usage have long been considered fundamental strategies in healthcare systems, and these approaches were traditionally viewed as the primary defense against bacterial resistance development. But recent studies reveal a surprising disconnect between antibiotic usage and resistance patterns, with socioeconomic factors showing stronger correlations than clinical drug use. Multiple factors beyond antibiotic consumption now influence resistance patterns, including agricultural antibiotic use, increasing urbanization, and the evolution of mobile genetic elements. Therefore, while antibiotic stewardship remains crucial for preventing side effects and reducing healthcare costs, its role in controlling bacterial resistance requires fundamental reassessment. This understanding necessitates a strategic shift in stewardship programs to focus on more attainable goals, such as patient safety and cost reduction, while developing new, comprehensive approaches to address antibiotic resistance that account for the complex interplay of biological, environmental, and socioeconomic factors.

## Introduction

Many pharmaceutical drugs are abused or misused: GLP-1 analogs, for instance, are now being widely promoted for weight loss, while other safer, cheaper drugs have been available for decades (Elmaleh-Sachs et al., 2023); nonsteroidal anti-inflammatory drugs reportedly resulted in “approximately 100,000 hospitalizations and 16,500 deaths each year” during the 1990s in the USA alone (Abramson and Weaver, 2005). Yet there are no documented stewardship campaigns for these or other misused medications. On the other hand, many antibiotic stewardship programs exist worldwide; using “antibiotic stewardship” as a search term in *PubMed* yielded 5,307 papers (as of August 5, 2025), the first one appearing in 1999 (Gould, 1999), and a record 732 published in 2024 after a three-year plateau of around 650/year. While clearly antibiotic abuse or misuse can lead to efficacy and safety issues for the affected patient, as with any other kind of drug, the main rationale for antibiotic stewardship campaigns is to somehow affect the growing prevalence of antibiotic resistant (AR) bacteria. From “there is an unequivocal link between antibiotic use and the devopment (*sic*) of resistance” (Gould, 1999); thru “patients in rural and undeserved communities face a disproportionate burden of antibiotic-resistant infections due to inappropriate antibiotic prescribing practices” (Nkemdirim Okere et al., 2025) (among the latest papers that the said *PubMed* search yielded), the notion of a strong direct correlation between the clinical use of antibiotics, and bacterial resistance, is prevalent within the stewardship literature. Yet, this notion is seemingly wrong.

The link usage-resistance makes sense from a Darwinian perspective; also, early evidence supported the existence of such a direct correlation. Recent analyses, however, fail to find this link, with many other factors having stronger correlation to AR prevalence than antibiotic usage. Perhaps the early studies were flawed; but it is also conceivable that, back then, there actually was a link that is no longer relevant due to biological and environmental changes. This will be discussed in following paragraphs. An inherent derivative of this supposed link, *i.e.*, that reducing antibiotic usage will result in diminished resistance, was proved mostly wrong (Salyers and Amábile-Cuevas, 1997; Heinemann et al., 2000), perhaps with the exception of some few, very particular cases (*e.g.*, Imeneo et al. (2025)) and will no longer be mentioned here. Aside, antibiotic stewardship campaigns are very much needed to diminish adverse effects, drug-drug interactions, and unnecessary spending, but not to address AR. (All discussion about “antibiotic stewardship” here, refers to nation-wide or even global efforts toward the rational use of antibiotics in human medicine; hospital stewardship programs may have some impact in modifying AR, in specific, limited antibiotic-germ combinations (Abejew et al., 2024).

## Old *vs*. new evidence on usage-resistance

There is some evidence that AR amongst bacterial pathogens emerged after the introduction of antibiotics into clinical use, as suggested by the lack of phenotypic AR in clinical isolates that predates this use —the Murray collection (Hughes and Datta, 1983). The very efficacy of early antibiotic treatments indicates that resistant pathogenic bacteria were rare. (However, it should be stated that antibiotic abuse did not and does not “cause” AR, nor that AR “emerges” because antibiotic usage (Amábile Cuevas, 2022).) Then came AR, and some studies that established a country-based link between usage and resistance: among others, Albrich et al. (2004) showed a correlation between outpatient consumption of antibiotics and penicillin- and macrolide-resistant *Streptococcus pneumoniae* and *S. pyogenes* in some developed countries; and van de Sande-Bruinsma et al. (2008) linked penicillin and fluoroquinolone usage to resistance in pneumococci and *Escherichia coli*, respectively, in European countries. But soon after, other studies showed that different socioeconomic factors are much more strongly associated to AR than antibiotic usage: income (gross domestic product, gross national income) and income inequality, corruption and other governance issues, health expenditure and infrastructure, and access to healthcare, all contribute significantly to the AR problem (Amábile-Cuevas and Lund-Zaina, 2024). Two striking examples can illustrate the point: the 50% reduction in antibiotic usage in Europe between 2008 and 2018 happening along a 17% increase in AR (Rahman et al., 2023); and the higher prevalence of AR in pathogens acquired in Mexico, compared to those acquired in the USA, for similar, paired infections (Berndtson et al., 2019), even when the USA consumes nearly four times more antibiotics than Mexico (Klein et al., 2018). The latter is also an example of this phenomenon affecting particularly more so the low and middle income countries (LMICs) where, closing a vicious circle, the economic burden of resistance becomes higher, and where lack of access to effective antibiotics is worse than antibiotic misuse (Laxminarayan et al., 2016). In any case, antibiotic stewardship does not appear to be of significant help in the countries where most (6.6 billion) people live, and could be encompassed into the “biomedical interventionism with which postcolonial medicine has been characterized”, along with other strategies to face AR (Haenssgen et al., 2020).

All of the research in the above paragraph is based on phenotypic AR as detected and defined by very dated methods (*i.e.*, disk-diffusion that was standardized by Bauer et al. in 1966, or serial dilution introduced by Fleming in 1928), so that a host of “non-canonical” AR phenotypes that also compromise the efficacy of antibiotic therapies are not detected (Amábile-Cuevas and Lund-Zaina, 2024). Even the supposedly simple notion of canonical resistance varies between guidelines (*e.g.*, CLSI, EUCAST) and changes over time, complicating the comparison and interpretation of resistance data; the changes in penicillin-resistance breakpoints for pneumococci in 2008-2009 is a dramatic example (Imöhl et al., 2014), but other, subtle changes may also affect the analyses of resistance trends. Furthermore, all of this research is based on routine AR data from clinical isolates, therefore representing the prevalence of “AR pathogens causing infections” rather than just the prevalence of AR. The latter issue must be stressed, as it may well be a reason for the major involvement of socioeconomic variables in the purported prevalence of AR in what should otherwise be a textbook example of evolution (AR) by (not-so-) natural selection (antibiotic usage). By measuring instead the prevalence of AR pathogens causing infection, we are including factors that affect the prevalence of pathogens and of infection, which may confound the role of antibiotic usage. Evidence of the usage-resistance link among the general bacterial population is scarce and contradictory: analyzing resistomes instead of AR phenotypes of isolated pathogens, a correlation between AR gene abundance (with the significant limitations of this approach (Jovanovic et al., 2021)) and antibiotic usage per country was found (Lee et al., 2023); but aminoglycoside-resistance genes are detected in the sewage of European countries, that have not used aminoglycosides for many years, as frequently as in Latin America or East Asia, where such drugs are used routinely (Munk et al., 2022). Phenotypic AR in commensal *Neisseria* spp. was related to antibiotic usage when comparing four countries (Kanesaka et al., 2025); but this was not found in commensal oral streptococci (Díaz-Mejía et al., 2002). Nevertheless, for the clinical purposes pertaining AR, perhaps we should stick to the prevalence of “AR pathogens causing infections” as this is, in the end, what we are trying to control. And this prevalence is not mainly driven by the clinical use of antibiotics.

## Non-clinical use of antibiotics: the persisting surrendering of public health to financial interests

Seventy two years ago, Barnett Stross stated before the British House of Commons that “if pigs are fed in this way [antibiotics as growth promoters], new types of bacteria may evolve and thrive which are resistant to the penicillin … [and] if there be migration of the bacteria to humans we may find ourselves in trouble” (Angus, 2019). Levy et al. (1976) showed this to be entirely true, measuring the spread of tetracycline-resistant bacteria among farm workers, their families and neighbors, after the introduction of tetracycline-supplemented feed for chickens. Decades later, we are still rediscovering this problem: an editorial piece in *Sci Am* of March, 2023, is entitled “To fight antimicrobial resistance, start with farm animals” (The Editors, 2023). But a brief note in *Nature*, a month before, was entitled “Antibiotic use in farming set to soar despite drug-resistance fears” (Reardon, 2023). With about 70% of the antibiotics produced worldwide going to some kind of agricultural use, this is a major contributor to AR prevalence, either as a direct source of resistant bacteria in foodstuff, or indirectly contributing to the AR gene pool in the environment (Amábile-Cuevas, 2021). Being more that double the clinical use, this could also be confounding the role of antibiotic clinical usage on AR prevalence, and should certainly be at the top of any antibiotic stewardship strategy (in a broad, so-called “One Health” perspective (Hibbard et al., 2024)), well above the use in humans. However, as antibiotic usage alone does not seems to correlate to AR prevalence in farm animals either (Smith et al., 2023), the role of reducing usage aiming to control AR is also doubtful.

## Biological and environmental changes: did we let the genie out of the bottle?

**Figure 1:**
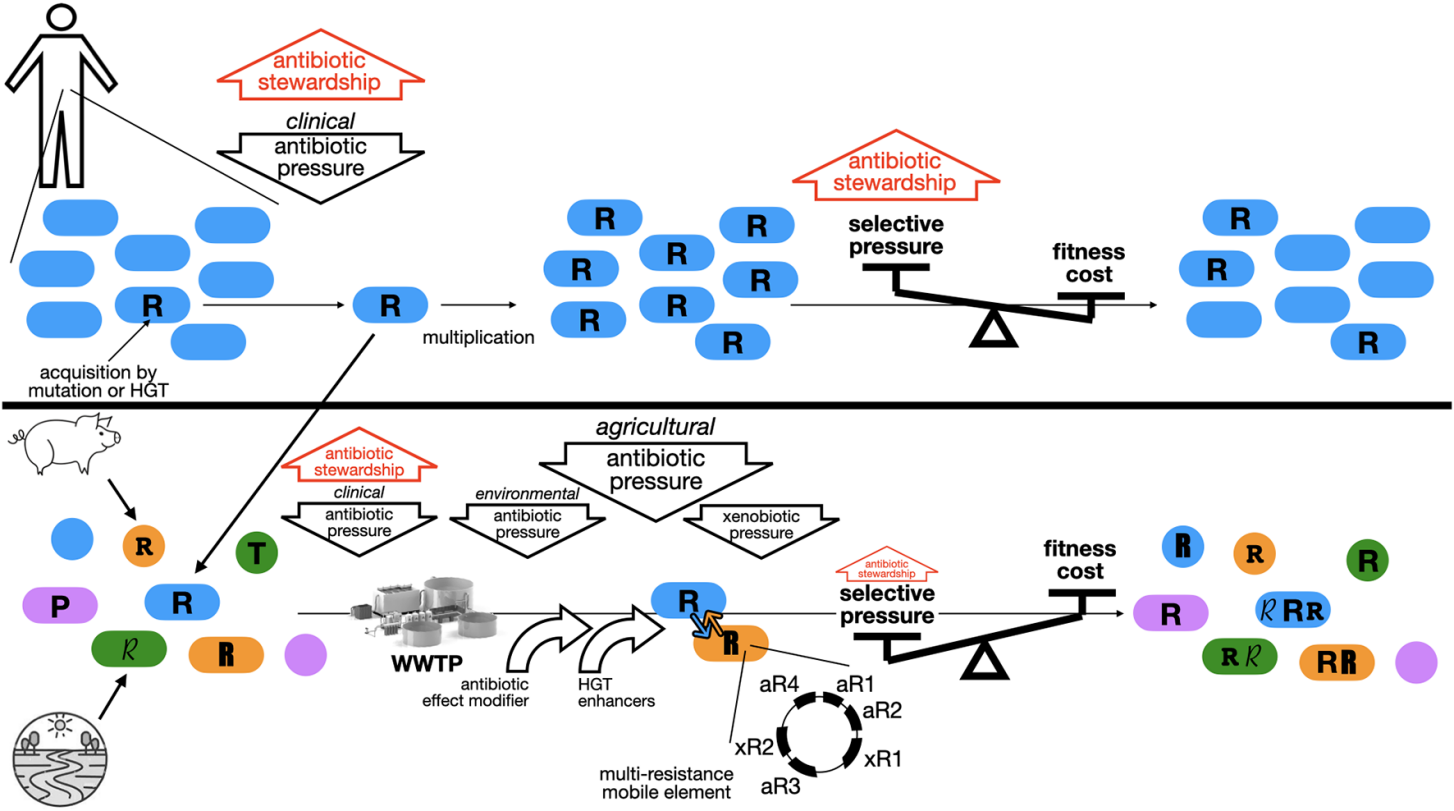
Supposed and actual relevance of antibiotic stewardship in modifying AR. TOP, the early notions of AR evolution and the role of stewardship: pathogenic bacteria (blue) isolated from an infected patient, mostly susceptible to an antibiotic, except for a very few that gained resistance through mutation or horizontal gene transfer (HGT), are exposed to the pressure of the administered antibiotic. Only the resistant one survives and, after multiplication, makes for a mostly resistant population. Then, a balance between the subsequent exposure to more antibiotic, and a purported fitness cost of the gained resistance, would either maintain or dilute the resistance determinant in the population. Antibiotic stewardship, by lifting the pressure, either in the early selection, or in the maintenance phase, would result in the dilution of the resistant organisms and recovery of antibiotic efficacy. BOTTOM, the actual scenario: the resistant variant selected by the clinical use of antibiotics above, joins a number of other resistant organisms selected for in food animals, and selected for, or anciently carrying resistance in the environment (with different fonts of “R” indicating different resistance determinants), along with other phenotypes, such as tolerance (T) or persistence (P) that diminish the efficacy of antibiotics. They coexist in many different scenarios under the selective pressures of antibiotics used clinically or agriculturally, or those released to the environment; some of this exposure occurs in gigantic “concentrators”, the wastewater treatment plants (WWTP), where commensal and pathogenic bacteria are mixed with antibiotics and other xenobiotics. Additionally, a number of chemical agents are capable of modifying the antibiotics’ effects, or increasing the rates of gene exchange (HGT enhancers), fostering the accretion of resistance genes (both towards antibiotics, aR; and towards xenobiotics, xR) in single genetic elements. With fitness costs usually negligible, the balance is almost always tipped to the pressure side, favoring the prevalence of multi-resistance in the global bacterial population. Antibiotic stewardship in the clinical side can only lift a little fraction of this pressure, to have also little effect on resistance prevalence.

What if antibiotic usage and resistance were actually linked together earlier, but something has changed? At least five relevant factors did change (or have potentially changed) in the last years: (a) the accretion of genes encoding AR and other traits that enable co-selection; (b) the massive environmental release of compounds that either select for AR, or facilitate horizontal gene transfer (HGT), many of them considered “emerging organic contaminants”; (c) the amounts and densities of human and animal populations exposed to antibiotics (and other xenobiotics), and the concentration of bacteria, antibiotics and xenobiotics in facilities such as wastewater treatment plants; (d) the evolution of AR plasmids and of HGT itself; and (e) the emergence of novel mechanisms of resistance, and of other phenotypes that reduce the efficacy of antibiotic treatments (Amábile-Cuevas and Lund-Zaina, 2024). While it is difficult to distinguish between “recently emerged” and “recently discovered”, particularly regarding the biological components of this equation, it is likely that multi-resistance plasmids and the roles of gene mobility elements and mechanisms, are continuously changing under the selective pressure of antibiotics and other xenobiotics; and that the amounts and diversity of such xenobiotics are significantly increasing over time. Hence, it is conceivable that, for instance, the use of penicillin was indeed the main driver of the increasing prevalence of penicillinase-producing *Staphylococcus aureus*, back in the early “antibiotic era”, when penicillinase plasmids, of limited mobility, carried only a few other resistance genes, and exposure to other relevant xenobiotics was sparse. But now, in a human population the double or triple than in the 1960’s (and a majority now crammed in urban areas), the selection and maintenance of multi-resistant enteric bacteria, carrying and exchanging a wide diversity of mobile genetic elements, exposed to many different antibiotics, within patients or food animals, and in the environment, along with chemical agents that modify their response to antibiotics (from triclosan in hand soap, to glyphosate in the soil) and/or their gene mobility (from noncaloric sweeteners to non-antibiotic medications), under a globally changing environment, make for a very complex scenario where the pressure exerted by clinically-used antibiotics is but a wee contributing factor (**Figure 1**). It could also be the reason for the proposed acceleration of the evolution of AR (Witzany et al., 2020), a particularly concerning possibility.

## Antibiotic stewardship: what for, then?

All of the above is not to say that antibiotic stewardship programs should disappear; but the objectives must shift from AR towards other relevant issues so that the programs are better tailored, and false expectations are avoided. For instance, trying to assess the cost-effectiveness of stewardship programs merely by measuring the economic impact of AR, as has been proposed (Roope et al., 2024), may completely miss the point. The “Political Declaration” after the 2024 High-level United Nations Meeting on Antimicrobial Resistance (UN, 2024a) still puts stewardship among the main strategies to face the AR problem: “prioritizing good antimicrobial stewardship”, “note the importance of improving the appropriate, prudent and responsible use of antimicrobials”, “investing in and strengthening stewardship programmes”, are examples of declarations and commitments in this document. (Curiously enough, a press release on the meeting, from the UN itself, does not even include the word “stewardship” (UN, 2024b).) The World Health Organization (WHO), however, seems to be quietly making the shift: in its guideline for stewardship programs in LMICs (WHO, 2019), among the eight aims of an antibiotic stewardship program, only three refer to AR (*i.e.*, “to reduce further emergence, selection and spread of AMR; to prolong the lifespan of existing antibiotics; [and] to limit the adverse economic impact of AMR”); and its “case study”, a stewardship program established after an outbreak of carbapenem-resistant *Klebsiella pneumoniae*, the reported achievement of the program was “a 60% decline in the use of carbapenems and vancomycin”, but no AR change was mentioned. So, what are the other five aims? Aside from the wordiness typical of these official documents, two are of great importance: “to improve quality of care and patient outcomes”, and “to save on unnecessary health-care costs”. The former should encompass the selection of the right drug and dosing, so that side-effects and drug-drug interactions are minimized; while the latter would be the result of the savings achieved by reducing expenditure both on unnecessary antibiotics, and on managing the side-effects of wrong treatments. These are no small aims, with 252 million antibiotic prescriptions for outpatients in 2023, in the USA alone (CDC, 2024), and up to 76% (191 millions)! of them being probably inappropriate (*e.g.* (Shively et al., 2018). But still aiming stewardship at reducing or controlling AR may condemn the efforts to miss the target. While the direct impact of stewardship on reducing immediate adverse effects of antibiotics is very relevant (Bauer et al., 2019), mid- to long-term side-effects of antibiotic usage, from disruption of the microbiota (Ramirez et al., 2020) to development of atopic dermatitis (Zhao et al., 2025) should be taken into account. As to the costs, antibiotic stewardship interventions do result in important savings, from €2575/mo in a single hospital department in Germany, to an estimated US$2.5 billion per year nationally in the USA (Naylor et al., 2017).

## Final considerations

It is difficult to depart from old, well-accepted notions that, in addition, seem to make sense, and replace them with counter-intuitive concepts based on complex data collections and analyses. But AR is the kind of “super-wicked” problem (Littmann et al., 2020) that calls for precisely this. AR has been named “a glocal syndemic”: glocal indicating the concurring role of local and global circumstances; syndemic meaning two or more health problems (*e.g*., malnutrition, infectious and noncommunicable diseases, climate change) concurring and interacting, and having common societal drivers (Ferrinho et al., 2023). Furthermore, stewardship programs aimed at patients and clinicians, sponsored by pharmaceutical companies and governments, look suspiciously like trying to pass the responsibility of AR onto consumers, very much as it is happening with climate change (Park, 2022). With the USA banning terms like “climate crisis”, “disparity”, “inequalities” and “socioeconomic”, from official documents (Yourish et al., 2025), it is unlikely that the role of socioeconomic disparities and inequalities, and of climate change, on the AR crisis will be accepted in that country any time soon (and also banning Diversity, Equity, and Inclusion notions, would likely deny the role of gender in AR (Jones et al., 2022)). This fortunately coincides with the USA’s withdrawal from the WHO, opening an invaluable opportunity for the latter to take a leading role (or to “get stuff done” as was also recommended to the UN (Singer, 2024)), for the rest of the planet, in shaping a globalized effort to harness AR. Only an extensive number of “national actions”, aside from stewardship, have been proved useful in reducing AR (Søgaard Jørgensen et al., 2025). Perhaps 10 million deaths per year in 2050 due to AR (O’Neil, 2016) was an exaggeration, but still 40 million deaths by 2050 (Naddaf, 2024) is an appalling perspective. And this figure is not going to diminish merely by stewardshipping antibiotics.

## Data Availability

The original contributions presented in the study are included in the article/supplementary material. Further inquiries can be directed to the corresponding author.
